# Assessing Wealth-Related Inequalities in Demand for Family Planning Satisfied in 43 African Countries

**DOI:** 10.3389/fgwh.2021.674227

**Published:** 2021-07-26

**Authors:** Franciele Hellwig, Carolina V. N. Coll, Cauane Blumenberg, Fernanda Ewerling, Caroline W. Kabiru, Aluisio J. D. Barros

**Affiliations:** ^1^International Center for Equity in Health, Federal University of Pelotas, Pelotas, Brazil; ^2^Postgraduate Program in Epidemiology, Federal University of Pelotas, Pelotas, Brazil; ^3^Population Dynamics and Reproductive Health Unit, African Population and Health Research Center, Nairobi, Kenya

**Keywords:** urban health, urban poor, informal settlement, contraception, family planning, Africa

## Abstract

**Background:** Around 80% of the African population lives in urban areas, and a rapid urbanization is observed in almost all countries. Urban poverty has been linked to several sexual and reproductive health risks, including high levels of unintended pregnancies. We aim to investigate wealth inequalities in demand for family planning satisfied with modern methods (mDFPS) among women living in urban areas from African countries.

**Methods:** We used data from 43 national health surveys carried out since 2010 to assess wealth inequalities in mDFPS. mDFPS and the share of modern contraceptive use were stratified by groups of household wealth. We also assessed the ecological relationship between the proportion of urban population living in informal settlements and both mDFPS and inequalities in coverage.

**Results:** mDFPS among urban women ranged from 27% (95% CI: 23–31%) in Chad to 87% (95% CI: 84–89%) in Eswatini. We found significant inequalities in mDFPS with lower coverage among the poorest women in most countries. In North Africa, inequalities in mDFPS were identified only in Sudan, where coverage ranged between 7% (95% CI: 3–15%) among the poorest and 52% (95% CI: 49–56%) among the wealthiest. The largest gap in the Eastern and Southern African was found in Angola; 6% (95% CI: 3–11%) among the poorest and 46% (95% CI: 41–51%) among the wealthiest. In West and Central Africa, large gaps were found for almost all countries, especially in Central African Republic, where mDFPS was 11% (95% CI: 7–18%) among the poorest and 47% (95% CI: 41–53%) among the wealthiest. Inequalities by type of method were also observed for urban poor, with an overall pattern of lower use of long-acting and permanent methods. Our ecological analyses showed that the higher the proportion of the population living in informal settlements, the lower the mDFPS and the higher the inequalities.

**Conclusion:** Our results rise the need for more focus on the urban-poorer women by public policies and programs. Future interventions developed by national governments and international organizations should consider the interconnection between urbanization, poverty, and reproductive health.

## Introduction

Population growth and dynamics pose many challenges to the achievement of the sustainable development agenda, particularly in low and middle-income contexts (1). Despite being one of the least urbanized places in the world, Africa has the fastest urban growth (2) and a positive annual urbanization rate is observed in almost all countries across the continent (3). Overall, urban areas usually have higher levels of coverage of health services than rural areas. However, urban poverty is increasing in many settings, especially in those experiencing a rapid urban transition, which may disproportionately affect people living in informal settlements and other vulnerable and marginalized groups (4–6). In several countries, despite the higher access to health care and availability of services generally found in urban areas, key maternal, and child health indicators for the urban poor present undesirably low coverage—similar to what is found for the rural poor populations (7–9).

Urban poverty has been linked to several sexual and reproductive health risks, including high levels of unintended pregnancies, and increased risk of sexually transmitted infections (10–12). Although the number of women in need of family planning using contraceptives is increasing worldwide, the demand for family planning satisfied with modern methods (mDFPS) is still low in several African countries, with little progress over time (4, 13–16). Comparing several interventions of the continuum of care, family planning coverage was one of the interventions with less progress overtime (15). Although specific programs like the Urban Reproductive Health Initiative have resulted in increased access to modern contraceptives for women living in urban areas of Kenya, Nigeria, and Senegal (13, 17), most policies and programs have failed to address the sexual and reproductive health needs of the most vulnerable urban populations (10). As a result, persistent high levels of unplanned pregnancies still being identified among urban women in sub-Saharan countries (4), and important inequalities in the coverage of mDFPS remain between and within countries across Africa (14, 16, 18). The West and Central Africa region presents the lowest average coverage of mDFPS, 33%, while average mDFPS reaches almost 60% in Eastern and Southern Africa (18). In most of African countries, coverage is lower among women who are poorer, less educated, and younger (16, 18).

Promoting family planning in urban contexts is critical to the development of healthy and productive urban populations, boosting economic growth, and ultimately the improvement of quality of life and achievement of the Sustainable Development Goals (10). Despite the abundant literature on national inequalities in mDFPS coverage, less is known about intra-urban inequalities. As most low- and middle-income countries are quickly becoming urban, efforts to achieve universal mDFPS require an understanding not only of urban-rural inequalities, but an understanding of intra-urban disparities in family planning coverage and the identification of population subgroups that are being left behind. In this study, we assessed between- and within-country wealth inequalities in mDFPS coverage among women 15–49 years of age living in urban areas of 43 African countries. We used data from national health surveys to perform the analyses, which had a particular emphasis on investigating whether urban poor women have lower mDFPS coverage in comparison to the other subgroups of the population.

## Materials and Methods

We used data from urban samples of Multiple Indicator Cluster Surveys (MICS) and Demographic and Health Surveys (DHS) carried out from 2010 to 2018 in African countries. All surveys that collected information on family planning for women aged 15–49 years were included. Forty-three surveys conducted across Africa were included in the analyses (Table 1). Analyses were based on 93,713 sexually active women irrespective of marital status, with a few exceptions. In Lesotho, Mauritania, and all surveys from the North African region—Algeria, Egypt, Sudan, and Tunisia—the samples were restricted to women who were married or in a union because the information on contraception was not collected for never-married women. Women were considered sexually active if they were married or living with a partner, or if they reported having had sexual intercourse in the month preceding the interview. Information on each survey sample is presented in Table 1.

**Table 1:** Countries included, sample characteristics, demand for family planning satisfied by modern methods (mDFPS), concentration index of inequality (CIX), and proportion of urban population living in informal settlements.

**Country**	**Year**	**Source**	**Women sample**	**Unweighted sample size**	**mDFPS % (95% CI)**	**CIX**	**Informal settlements (%)**
**North Africa**					69.6 (40.6; 98.5)		
Algeria	2012	MICS	Currently married	8,086	73.4 (71.7; 75.0)	0.2	NA
Egypt	2014	DHS	Currently married	6,478	81.4 (80.0; 82.7)	0.7	10.6
Sudan	2014	MICS	Currently married	1,448	42.8 (39.6; 46.2)	17.6	91.6
Tunisia	2018	MICS	Currently married	2,908	80.7 (79.0; 82.3)	0.4	8.2
**Eastern and Southern Africa**					67.3 (59.2; 75.4)		
Angola	2015	DHS	Sexually active	3,424	34.1 (30.4; 38.1)	26.3	55.5
Burundi	2016	DHS	Sexually active	1,098	47.6 (42.7; 52.6)	5.8	48.6
Comoros	2012	DHS	Sexually active	784	37.6 (32.9; 42.6)	0.1	69.6
Eswatini	2014	MICS	Sexually active	529	86.8 (84.0; 89.2)	1.4	32.7
Ethiopia	2016	DHS	Sexually active	1,567	77.2 (73.3; 80.6)	−3.1	65.9
Kenya	2014	DHS	Sexually active	2,642	75.7 (73.3; 78.0)	2.3	56.0
Lesotho	2018	MICS	Currently married	940	78.9 (75.4; 82.0)	1.3	53.6
Madagascar	2018	MICS	Sexually active	2,169	60.7 (58.1; 63.3)	−6.2	61.2
Malawi	2015	DHS	Sexually active	2,587	75.0 (73.0; 76.9)	−1.6	66.7
Mozambique	2015	DHS	Sexually active	1,456	61.1 (57.2; 64.8)	8.0	80.3
Namibia	2013	DHS	Sexually active	2,092	82.7 (80.4; 84.7)	1.6	39.4
Rwanda	2014	DHS	Sexually active	1,233	64.9 (61.7; 68.0)	−0.4	53.2
South Africa	2016	DHS	Sexually active	2,083	76.6 (74.0; 79.0)	0.3	26.3
Tanzania	2015	DHS	Sexually active	1,679	53.8 (50.6; 57.0)	0.2	50.7
Uganda	2016	DHS	Sexually active	1,805	57.4 (54.6; 60.1)	3.4	47.5
Zambia	2018	DHS	Sexually active	2,199	69.1 (66.6; 71.5)	−0.1	54.6
Zimbabwe	2015	DHS	Sexually active	2,192	85.7 (83.3; 87.9)	1.8	25.1
**West and Central Africa**					43.3 (38.9; 47.8)		
Benin	2017	DHS	Sexually active	2,755	27.4 (25.0; 29.8)	7.4	59.6
Burkina Faso	2010	DHS	Sexually active	2,099	56.3 (52.9; 59.5)	7.0	65.8
Central African Republic	2010	MICS	Sexually active	1,619	38.2 (33.7; 42.8)	18.1	95.9
Cameroon	2018	DHS	Sexually active	2,549	43.9 (41.6; 46.3)	6.4	33.7
Chad	2014	DHS	Sexually active	1,100	26.5 (22.7; 30.7)	8.0	88.2
Congo Brazzaville	2014	MICS	Sexually active	1,737	41.4 (38.9; 43.9)	5.2	46.9
Congo Democratic Republic	2017	MICS	Sexually active	2,834	36.6 (33.0; 40.2)	21.8	79.1
Cote dIvoire	2016	MICS	Sexually active	1,597	38.5 (35.1; 42.1)	10.5	59.2
Gabon	2012	DHS	Sexually active	2,540	41.4 (38.0; 44.9)	7.3	37.0
Gambia	2018	MICS	Sexually active	1,750	39.9 (36.6; 43.4)	8.6	27.1
Ghana	2017	MICS	Sexually active	2,329	33.8 (31.3; 36.4)	−0.7	30.4
Guinea	2018	DHS	Sexually active	1,128	37.7 (33.0; 42.7)	12.2	50.1
Guinea Bissau	2014	MICS	Sexually active	1,509	61.0 (57.3; 64.7)	1.7	74.4
Liberia	2013	DHS	Sexually active	1,667	41.3 (36.7; 46.0)	1.7	65.7
Mali	2018	DHS	Sexually active	1,103	48.7 (44.2; 53;2)	9.4	47.2
Mauritania	2015	MICS	Currently married	2,340	42.2 (38.7; 45.8)	6.5	79.9
Niger	2012	DHS	Sexually active	1,121	50.3 (46.0; 54.6)	6.8	81.7
Nigeria	2018	DHS	Sexually active	5,310	35.1 (33.2; 37.1)	7.2	53.9
Sao Tome and Principe	2014	MICS	Sexually active	884	47.3 (43.3; 51.3)	2.1	86.6
Senegal	2017	DHS	Sexually active	2,289	65.8 (62.7; 68.7)	3.0	29.5
Sierra Leone	2017	MICS	Sexually active	2,821	57.8 (54.9; 60.6)	3.0	59.8
Togo	2017	MICS	Sexually active	1,233	42.6 (38.5; 46.7)	4.9	53.0

### Outcome

mDFPS coverage was defined as the proportion of women in need of contraception that were using (or whose partner was using) a modern contraceptive method. A woman was considered in need of contraception if she was fecund and did not want to become pregnant within the next 2 years, or if she was unsure about whether or when she wants to become pregnant. Pregnant women with a mistimed or unplanned pregnancy are also considered in need of contraception. Methods were classified as modern if they are medical procedures or technological products (19), including oral contraceptive pills, injections, male and female condoms, intrauterine devices (IUD), spermicides, implants, and sterilization (female or male). These methods were further classified as short-acting reversible contraceptives (oral contraceptive pills, male and female condoms, injectables, diaphragms, spermicidal agents, and emergency contraception), long-acting reversible contraceptives (IUD and implants), and permanent methods (male and female sterilization). Regarding having their demand for family planning satisfied by modern methods, women in need of contraception and using a modern method were classified as yes, while those in need of contraception but not using a method other than those classified as modern, were classified as no.

### Stratification

#### Wealth Index

Household asset scores, generated through principal component analysis (PCA), were available in the DHS and MICS surveys. The PCA includes variables on household assets, building materials, and utilities like water and electricity, which are adjusted for the place of residence (20). DHS and MICS carried out separate PCA in urban and rural households as relevant assets may vary between them; both PCA are later combined into a single score using a scaling procedure to allow comparability between urban and rural households (21). The first component of the PCA, a continuous variable, was used to classify households into three groups of wealth, with the first group (T1) representing the poorest 1/3 of all families and the third one (T3) representing the wealthiest 1/3 of all families.

### Statistical Analyses

The coverage of mDFPS and the share of the mDFPS by short-acting, long-acting, and permanent contraceptive methods were calculated for each survey and organized according to the UNICEF regions (North Africa, Eastern and Southern Africa, and West and Central Africa) (22), considering all urban women and according to wealth groups. We also calculated the concentration index of inequality (CIX) for mDFPS, a complex relative inequality measure according to wealth index (23, 24). It ranges from −100 and +100, where a CIX equals to zero represents equal coverage of mDFPS according to the levels of wealth groups. Positive CIX values indicate a pro-rich scenario (i.e., higher mDFPS coverage among the wealthier), while negative values indicate a pro-poor scenario (24).

The proportion of the urban population living in informal settlements for the years of all surveys analyzed was also described. According to the UN-Habitat, a household is defined as an informal settlement if it lacks one or more basic conditions, including access to improved water, access to sanitation, sufficient living area, housing durability, security of tenure, and housing affordability (25). Representing, therefore, an important proxy of extreme poverty. These information were obtained from the World Bank Open Data (26).

The relationship between the proportion of the population living in informal settlements and both the mDFPS coverage and CIX were analyzed using restricted cubic splines to account for their non-linear relationship. The splines were generated for the informal settlement variable using from three to seven knots, and then regressed on the mDFPS coverage and CIX estimates. The final number of knots to be used was estimated based on the quality of adjustment of the models, using the smallest values of the Akaike's (AIC) and Bayesian Information Criteria (BIC). The location of the knots was defined based on Harrell's recommendation (27).

All analyses were performed using Stata software version 16.1 (StataCorp LLC, College Station, TX) and adjusted for the sample design, including sample weights, clusters, and strata. All analyses relied on publicly available anonymized databases. The institutions and national agencies in each country obtained ethics approval for the surveys.

## Results

### mDFPS Coverage and Share of Modern Contraceptive use Among Urban Women

Average urban mDFPS coverage among North African countries was 69.6% (95% CI: 40.6–98.5%), 67.4 (96% CI: 59.2–75.4%) for Eastern and Southern Africa countries, and 43.3% (95% CI: 38.9–47.8%) for countries from the West and Central Africa region (Table 1). At country level, the coverage of mDFPS among urban women ranged from 26.5% (95% CI: 22.7–30.7%) in Chad to 86.8% (95% CI: 84.0–89.3%) in Eswatini (Table 1). Countries in North Africa presented a high coverage of mDFPS (>70%), except for Sudan, where only 42.8% (95% CI: 39.6–46.2%) of urban women had their need for family planning satisfied by modern methods (Table 1).

**Figure 1:**
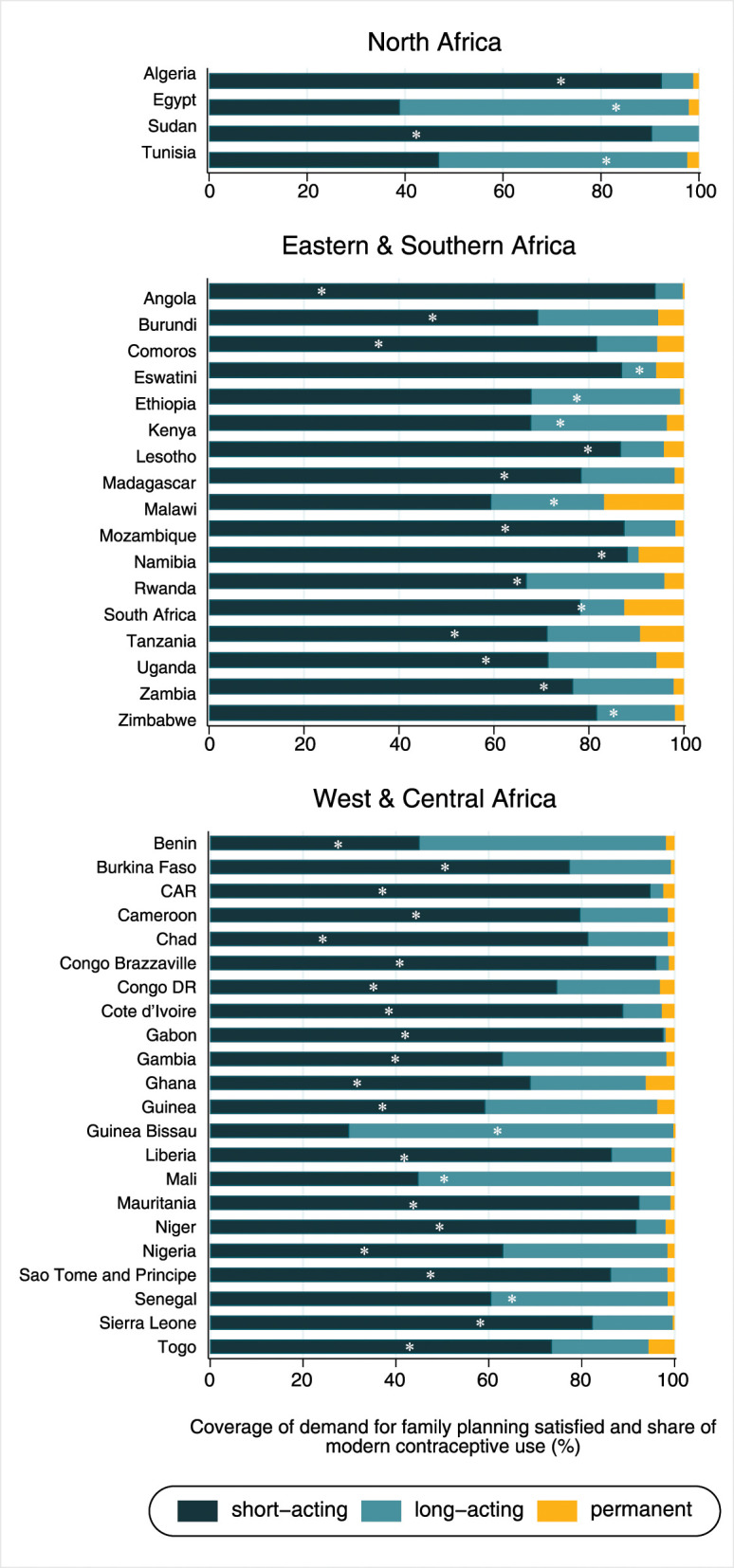
Share of modern contraceptive use and coverage of demand for family planning satisfied by modern methods (white dot meaning of “*”) according to world region.

According to the type of method, mDFPS in Algeria and Sudan was mainly achieved with short-acting reversible methods. Long-acting reversible methods were the most used in Egypt and Tunisia (Figure 1). In Eastern and Southern Africa, nine out of the 17 countries included in the analyses presented <75% of mDFPS coverage. Among the countries in the region, the lowest mDFPS coverage was found in Angola (34.1%; 95% CI: 30.4–38.1%). West and Central Africa presented the lowest mDFPS coverage across the African regions. mDFPS coverage reached 50% in only five out of the 22 countries: Senegal (65.8%; 95% CI: 62.7–68.7%), Guinea Bissau (61.0%; 95% CI: 57.3–64.7%), Sierra Leone (57.8%; 95% CI: 54.9–60.6%), Burkina Faso (56.3%; 95% CI: 52.9–59.5%), and Niger (50.3%; 95% CI: 46.0–54.6%). According to the share of method, short-acting reversible contraceptives were the most used in most countries. Long-acting methods were predominant only in Benin, Guinea Bissau, and Mali (mDPFS coverage of 53.0, 70.0, and 54.3%, respectively) (Figure 1).

### Wealth Inequalities in mDFPS

**Figure 2:**
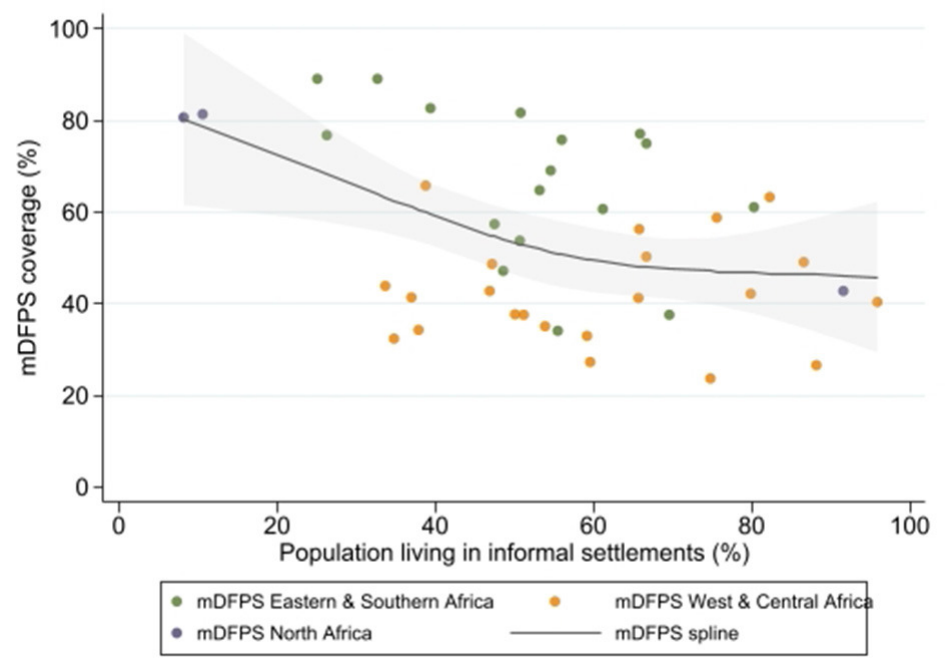
Cubic spline model showing the relationship between the coverage of demand for family planning satisfied by modern methods and the proportion of the population living in informal settlements for each region.

**Figure 3:**
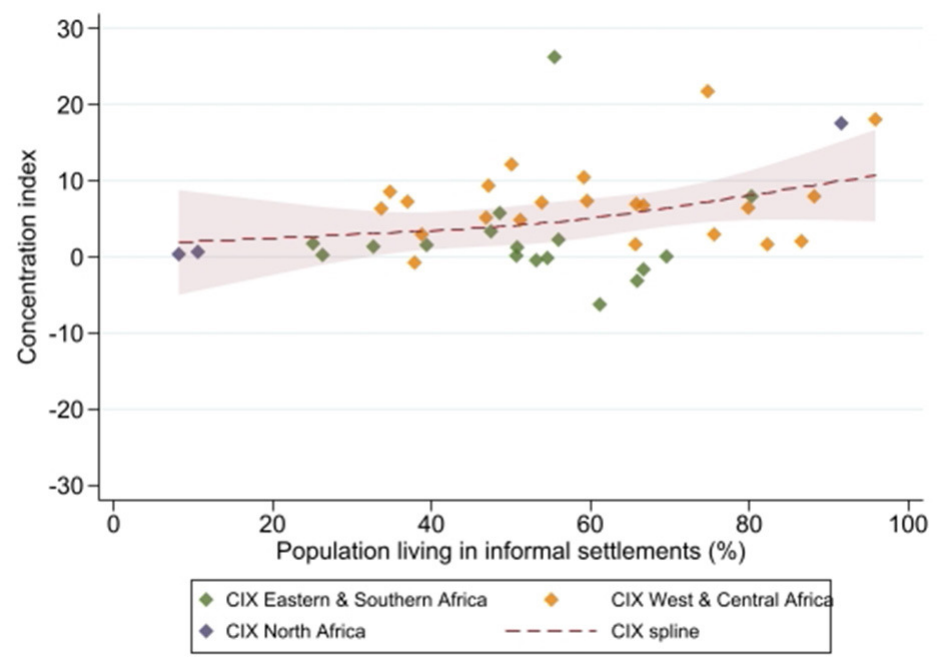
Cubic spline model showing the relationship between the concentration index and the proportion of the population living in informal settlements for each region.

The mDFPS coverage was also evaluated according to the proportion of people living in informal settlements. After testing different number of knots for the restricted cubic splines, the best model was obtained using three knots. An inverse relationship between the mDFPS coverage and the proportion of the population living in informal settlements was observed. The coverage reduced from 80% for the countries in which 10% of the population was living in informal settlements to approximately 45% for countries in which over 80% of the population was living in informal settlements (Figure 2). In contrast, a direct relationship between the relative inequalities (measured by the CIX) and the proportion of the population living in informal settlements was identified. The CIX increased from zero for countries with <20% to 10 for those with more than 90% of the population living in informal settlements, respectively (Figure 3).

**Figure 4:**
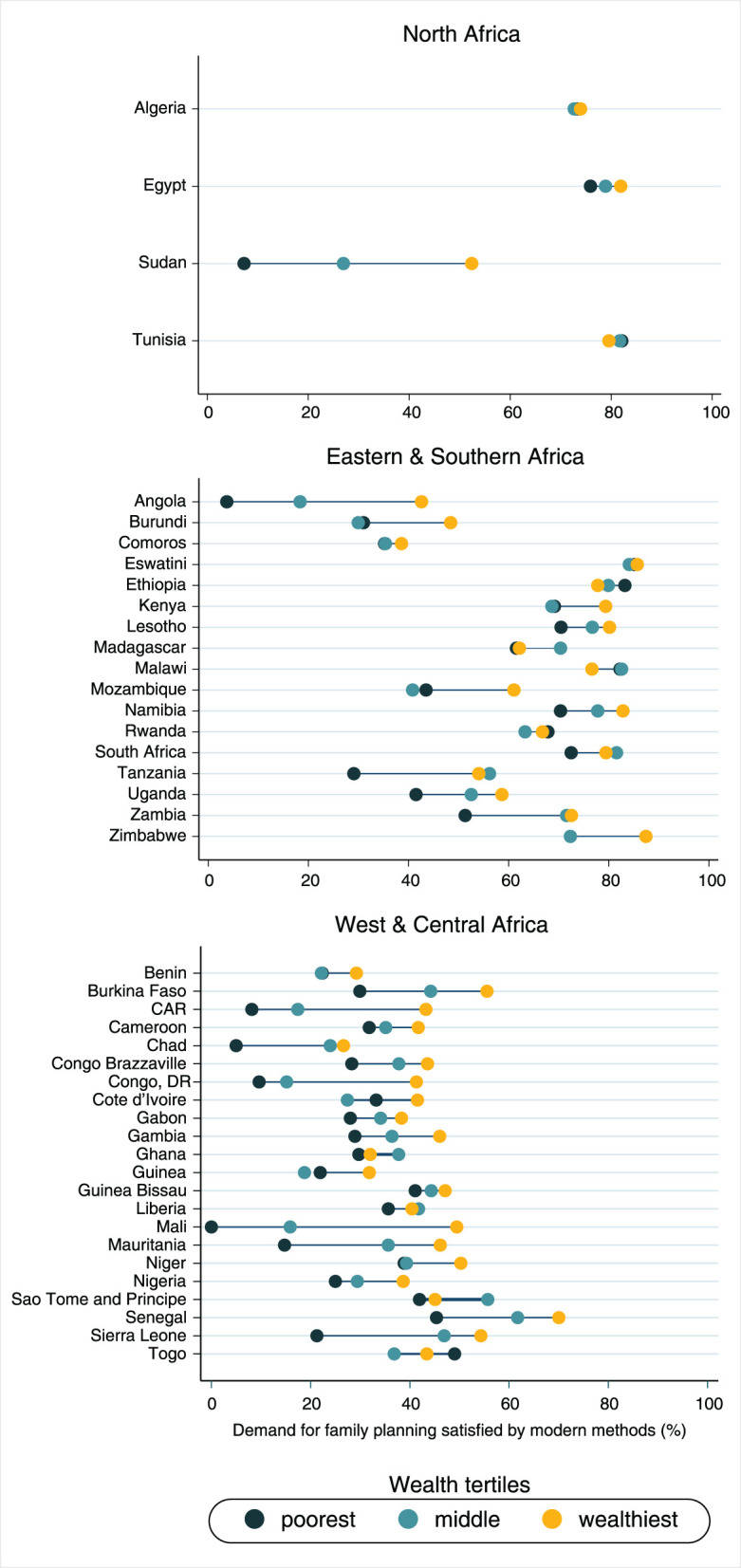
Demand for family planning satisfied by modern methods according to world region and wealth groups.

The analysis stratified in terms of wealth groups showed that mDFPS is, in general, much lower among the poorest women compared to the wealthiest (Figure 4). In North Africa, marked wealth inequalities were found only in Sudan, where mDFPS among the poorest was 7.3% (95% CI: 3.4–15.0%) and 52.4% (95% CI: 48.8–55.9%) among the wealthiest. The largest gap in the Eastern and Southern African region was found in Angola, where mDFPS ranged between 5.5% (95% CI: 2.6–11.4%) among the poorest and 45.9% (95% CI: 41.1–5.8%) among the wealthiest. In Tanzania and Zambia, a bottom pattern of inequality was observed, in which a much lower mDFPS coverage was found among the poorest women compared to the other wealth groups. mDFPS coverage gaps between richer and poorer women were virtually null in Eswatini and very small in Comoros, Rwanda, and South Africa. In West and Central Africa, huge gaps were found for almost all countries, especially in Central African Republic (CAR) and Mauritania, where mDFPS was, respectively, 10.1% (95% CI: 6.5–17.4%) and 14.7% (95% CI: 7.2–27.7%) for the poorest, and 44.4% (95% CI: 39.0–49.8%) and 46.1% (95% CI: 41.9–50.4%), respectively, for the middle and wealthiest groups (Figure 4). Detailed information on the sample size and 95% confidence intervals is presented in the Supplementary Material. Analyzing the CIX estimates, we also found a high level of wealth inequality in mDFPS in Sudan (CIX = 17.6), Angola (CIX = 26.3), the Democratic Republic of the Congo (CIX = 21.8), and Central African Republic (CIX = 18.1) (Table 1).

**Figure 5:**
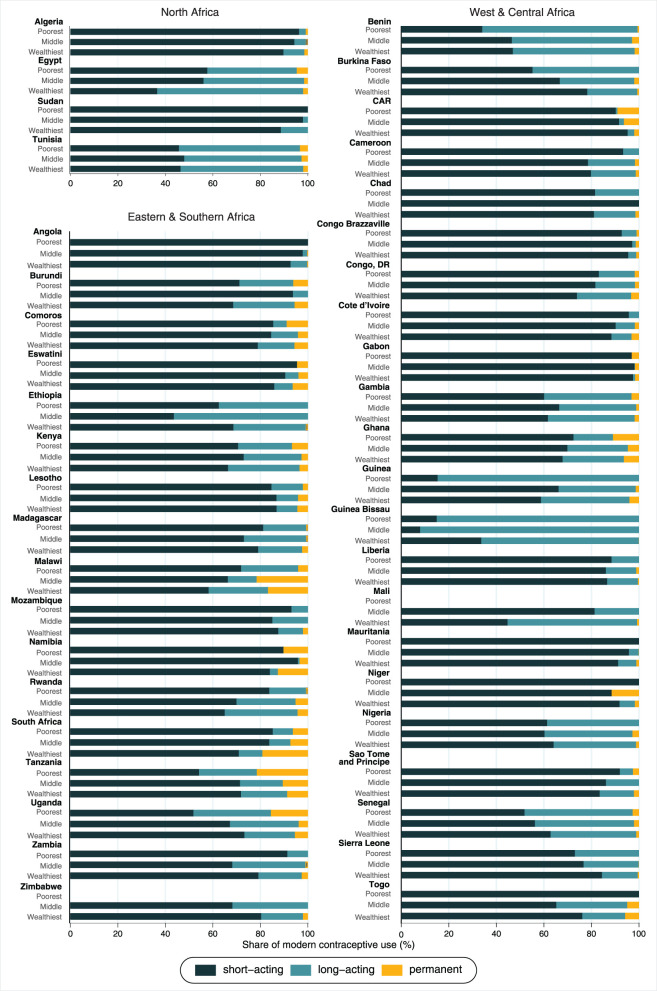
Share of modern contraceptive use according to wealth groups*. *In Mali and Zimbabwe, the bars related to the poorest are not presented because we have no observation on modern contraceptive use among women from the poorest wealth tertile.

Considering the share of modern contraceptive use according to wealth groups (Figure 5 and Table 2), in North Africa the proportion of women using long-acting reversible methods positively increased with wealth in almost all countries included. The only exception was Tunisia, where the share of long-acting reversible methods was similar across all groups of wealth and higher than the share of the other methods. Countries from West and Central Africa presented varied patterns of contraceptive methods mix. Higher share of long-acting methods among the wealthiest was found in the Democratic Republic of the Congo, Ghana, Mali, Mauritania, and Togo. In Eastern and Southern Africa, results on the association between the type of methods and wealth status were mixed. The share of long-acting reversible methods was lower among the urban poor than the wealthiest in Angola, Burundi, Comoros, Eswatini, Kenya, Namibia, Rwanda, and Zambia. On the other hand, the share of permanent methods (sterilization) was markedly higher among the poorest as compared to the wealthiest in Kenya (6.6 vs. 3.4%), Tanzania (21.4 vs. 8.7%), Uganda (15.5 vs. 5.4%), and Central African Republic (9.0 vs. 2.0%).

**Table 2:** Demand for family planning satisfied by modern methods (mDFPS), share of each type of contraceptive method, according to wealth groups.

**Country**	**Wealth groups**	**mDFPS**		**SARC**	**LARC**	**PERM**
		**% (95% CI)**	***N***			
**NORTH AFRICA**						
Algeria (2012)	Poorest	73.3 (69.4; 76.8)	1,385	96.3	2.8	0.9
	Middle	72.7 (70.0; 75.2)	2,694	94.3	4.9	0.8
	Wealthiest	73.9 (71.9; 75.9)	3,704	89.7	8.8	1.5
Egypt (2014)	Poorest	75.9 (67.7; 82.5)	221	57.7	37.7	4.7
	Middle	78.9 (74.5; 82.7)	436	56.1	42.4	1.6
	Wealthiest	81.9 (80.4; 83.3)	4,526	36.6	61.5	2.0
Sudan (2014)	Poorest	7.3 (3.4; 15.0)	67	100.0	0.0	0.0
	Middle	27.0 (21.9; 32.7)	451	97.9	2.1	0.0
	Wealthiest	52.4 (48.8; 55.9)	999	88.7	11.3	0.0
Tunisia (2018)	Poorest	82.1 (76.8; 86.4)	309	45.7	51.0	3.3
	Middle	81.7 (79.2; 83.9)	1,273	48.0	49.4	2.6
	Wealthiest	79.5 (76.9; 81.9)	1,493	46.4	51.7	1.9
**EASTERN AND SOUTHERN AFRICA**						
Angola (2015)	Poorest	5.5 (2.6; 11.4)	188	100.0	0.0	0.0
	Middle	21.0 (17.5; 25.0)	1,575	97.9	1.9	0.1
	Wealthiest	45.9 (41.1; 50.8)	2,213	92.8	7.1	0.1
Burundi (2016)	Poorest	30.3 (20.9; 41.8)	18	71.3	22.7	6.1
	Middle	30.6 (12.9; 56.8)	25	93.8	6.2	0.0
	Wealthiest	48.9 (43.9; 53.9)	575	68.7	25.8	5.5
Comoros (2012)	Poorest	33.6 (20.0; 50.6)	100	85.5	5.6	8.9
	Middle	38.5 (29.5; 48.3)	197	84.6	11.2	4.1
	Wealthiest	38.4 (33.0; 44.1)	318	79.0	15.4	5.6
Eswatini (2014)	Poorest	81.0 (65.9; 90.4)	21	95.5	0.0	4.5
	Middle	87.8 (79.3; 93.1)	139	90.5	5.6	3.9
	Wealthiest	86.8 (83.1; 89.8)	607	85.9	7.7	6.3
Ethiopia (2016)	Poorest	83.2 (67.4; 92.2)	37	62.6	37.4	0.0
	Middle	79.9 (65.5; 89.3)	24	43.6	56.4	0.0
	Wealthiest	76.9 (73.0; 80.4)	1,062	68.7	30.4	0.8
Kenya (2014)	Poorest	68.9 (63.1; 74.1)	241	70.7	22.7	6.6
	Middle	69.7 (64.3; 74.6)	537	73.1	24.4	2.6
	Wealthiest	78.0 (75.0; 80.7)	2,156	66.4	30.1	3.4
Lesotho (2018)	Poorest	70.5 (56.8; 81.2)	43	84.7	13.2	2.0
	Middle	76.7 (68.1; 83.6)	361	86.8	9.1	4.1
	Wealthiest	80.2 (76.3; 83.5)	892	86.9	8.8	4.3
Madagascar (2018)	Poorest	61.8 (53.5; 69.4)	197	81.3	18.0	0.7
	Middle	70.4 (64.6; 75.6)	248	73.1	26.2	0.7
	Wealthiest	59.0 (55.9; 62.0)	1,494	79.2	18.5	2.4
Malawi (2015)	Poorest	83.7 (71.1; 91.5)	61	72.0	24.0	4.0
	Middle	80.5 (73.7; 85.9)	203	66.4	12.1	21.5
	Wealthiest	74.2 (71.8; 76.5)	2,011	58.3	25.0	16.7
Mozambique (2015)	Poorest	50.6 (36.3; 64.7)	42	93.1	6.9	0.0
	Middle	42.4 (33.7; 51.6)	128	85.1	14.9	0.0
	Wealthiest	64.3 (60.6; 67.9)	879	87.6	10.4	2.0
Namibia (2013)	Poorest	74.2 (65.4; 81.4)	161	89.8	0.0	10.2
	Middle	81.9 (78.5; 84.9)	714	95.9	0.6	3.5
	Wealthiest	84.1 (81.1; 86.7)	1,338	84.1	3.4	12.6
Rwanda (2014)	Poorest	64.3 (56.4; 71.4)	75	83.8	15.4	0.8
	Middle	64.4 (53.2; 74.2)	77	70.0	24.8	5.1
	Wealthiest	65.0 (61.5; 68.4)	801	65.1	30.6	4.3
South Africa (2016)	Poorest	73.7 (67,6; 79.0)	564	85.3	8.4	6.3
	Middle	76.7 (72.5; 80.4)	884	83.8	8.9	7.3
	Wealthiest	77.8 (74.2; 81.1)	1,208	71.0	9.9	19.1
Tanzania (2015)	Poorest	32.4 (22.4; 44.3)	105	54.2	24.3	21.4
	Middle	53.4 (43.2; 63.4)	181	71.5	18.0	10.5
	Wealthiest	55.1 (51.9; 58.3)	1,755	71.9	19.4	8.7
Uganda (2016)	Poorest	40.9 (31.3; 51.2)	154	51.9	32.6	15.5
	Middle	52.6 (45.8; 59.4)	260	67.2	28.9	3.8
	Wealthiest	59.6 (56.4; 62.7)	1,673	73.3	21.3	5.4
Zambia (2018)	Poorest	46.7 (32.4; 61.5)	41	91.4	8.6	0.0
	Middle	68.0 (63.2; 72.4)	634	68.3	30.8	1.0
	Wealthiest	69.9 (67.0; 72.8)	1,919	79.2	18.3	2.5
Zimbabwe (2015)	Poorest	NO	NO	NO	NO	NO
	Middle	73.2 (58.8; 84.0)	60	68.3	31.7	0.0
	Wealthiest	86.1 (83.8; 88.2)	1,801	80.4	17.6	2.0
**WEST AND CENTRAL AFRICA**						
Benin (2017)	Poorest	22.7 (17.8; 28.5)	428	34.1	65.3	0.6
	Middle	24.2 (19.9; 29.0)	523	46.5	50.6	2.9
	Wealthiest	29.5 (26.7; 32.5)	1,687	47.0	51.2	1.8
Burkina Faso (2010)	Poorest	33.1 (21.7; 47.0)	32	55.2	44.8	0.0
	Middle	45.3 (37.3; 53.5)	109	66.7	31.3	2.0
	Wealthiest	57.4 (53.9; 60.8)	1,685	78.3	21.0	0.7
Central African Republic (2010)	Poorest	10.8 (96.5; 17.4)	98	90.2	0.7	9.0
	Middle	18.2 (14.5; 22.6)	352	91.6	2.1	6.3
	Wealthiest	44.4 (39.0; 49.8)	1,564	95.2	2.8	2.0
Cameroon (2018)	Poorest	33.6 (21.9; 47.8)	76	93.4	6.6	0.0
	Middle	39.4 (35.6; 43.4)	842	78.6	19.8	1.7
	Wealthiest	46.6 (43.8; 49.4)	1,722	79.8	18.8	1.4
Chad (2014)	Poorest	7.5 (2.0; 24.4)	67	81.5	18.5	0.0
	Middle	21.9 (5.3; 58.4)	28	100.0	0.0	0.0
	Wealthiest	27.8 (24.0; 31.9)	1,048	81.1	17.4	1.5
Congo Brazzaville (2014)	Poorest	28.6 (20.3; 38.6)	282	92.8	6.2	1.0
	Middle	40.9 (36.7; 45.1)	1,475	97.2	1.5	1.3
	Wealthiest	44.5 (40.4; 48.6)	1,420	95.5	3.3	1.1
Congo Democratic Republic (2017)	Poorest	11.5 (5.7; 22.0)	246	83.1	15.1	1.8
	Middle	17.0 (12.6; 22.5)	724	81.7	16.7	1.7
	Wealthiest	43.2 (39.0; 47.5)	3,055	73.9	22.8	3.3
Cote d'Ivoire (2016)	Poorest	32.3 (19.7; 48.0)	44	95.8	4.2	0.0
	Middle	28.9 (24.5; 33.8)	669	90.2	8.1	1.7
	Wealthiest	43.2 (38.9; 47.6)	1,433	88.4	8.5	3.1
Gabon (2012)	Poorest	34.9 (29.8; 40.3)	740	97.0	0.0	3.0
	Middle	39.2 (33.8; 44.8)	1,285	98.1	0.2	1.7
	Wealthiest	47.0 (40.2; 54.0)	1,366	97.5	0.9	1.6
Gambia	Poorest	29.1 (22.2; 37.1)	387	60.1	36.8	3.1
	Middle	36.5 (31.6; 41.7)	905	66.5	32.4	1.1
	Wealthiest	45.6 (41.5; 49.7)	1,293	61.7	36.5	1.8
Ghana (2017)	Poorest	30.3 (22.9; 39.0)	280	72.4	16.6	10.9
	Middle	36.8 (31.9; 41.9)	857	69.9	25.4	4.7
	Wealthiest	32.7 (29.3; 36.3)	1,430	67.9	25.8	6.4
Guinea (2018)	Poorest	19.0 (6.2; 45.5)	13	15.3	84.7	0.0
	Middle	26.3 (17.7; 37.3)	158	66.2	32.5	1.3
	Wealthiest	39.8 (34.7; 45.2)	968	58.8	37.2	4.0
Guinea Bissau (2018)	Poorest	59.4 (44.2; 72.9)	57	14.9	85.1	0.0
	Middle	57.9 (50.8; 64.6)	223	7.9	92.1	0.0
	Wealthiest	61.6 (57.1; 65.8)	1,478	33.7	66.3	0.1
Liberia (2013)	Poorest	36.0 (21.7; 46.0)	168	88.5	11.5	0.0
	Middle	43.5 (37.4; 49.8)	817	86.1	12.8	1.1
	Wealthiest	40.7 (34.6; 47.0)	1,530	86.6	13.0	0.4
Mali (2018)	Poorest	0.0	1	100.0	0.0	0.0
	Middle	16.4 (6.2; 36.5)	15	81.3	18.7	0.0
	Wealthiest	49.2 (44.7; 53.7)	955	44.7	54.5	0.8
Mauritania (2015)	Poorest	14.7 (7.2; 27.7)	57	100.0	0.0	0.0
	Middle	35.7 (30.5; 41.1)	709	95.8	4.1	0.1
	Wealthiest	46.1 (41.9; 50.4)	1,591	91.2	7.7	1.1
Niger (2012)	Poorest	38.9 (17.7; 65.2)	5	100.0	0.0	0.0
	Middle	39.3 (20.6; 61.9)	16	88.5	0.0	11.5
	Wealthiest	50.6 (46.1; 55.1)	664	91.9	6.4	1.7
Nigeria (2018)	Poorest	25.6 (19.4; 33.0)	308	61.4	38.6	0.0
	Middle	29.2 (26.3; 32.4)	1,601	60.3	37.0	2.7
	Wealthiest	38.0 (35.8; 40.3)	4,314	64.1	34.7	1.2
Sao Tome and Principe (2014)	Poorest	42.4 (34.7; 50.6)	253	92.0	5.5	2.5
	Middle	53.7 (47.5; 59.7)	288	86.1	13.9	0.0
	Wealthiest	45.9 (39.9; 52.0)	432	83.5	14.4	2.1
Senegal (2017)	Poorest	45.8 (36.7; 55.1)	108	51.9	45.5	2.6
	Middle	61.6 (56.8; 66.1)	806	56.3	41.7	2.1
	Wealthiest	69.1 (65.2; 72.7)	1,649	62.8	36.0	1.1
Sierra Leone (2017)	Poorest	34.1 (20.9; 50.4)	48	73.0	27.0	0.0
	Middle	52.8 (48.7; 56.9)	964	76.7	23.2	0.1
	Wealthiest	60.2 (56.9; 63.3)	2,485	84.5	15.0	0.5
Togo (2017)	Poorest	49.0 (5.6; 94.0)	4	100.0	0.0	0.0
	Middle	37.0 (30.7; 43.7)	388	65.2	29.9	4.9
	Wealthiest	44.6 (40.1; 49.2)	1,041	76.2	18.0	5.8

*SARC, short-acting reversible contraceptives; LARC, long-acting revesible contraceptives; PERM, permanent contraceptives*.

## Discussion

We reported on surveys from 43 African countries, analyzing a sample of 93,713 women to assess between-country and within-country wealth inequalities in mDFPS coverage among urban populations. Our findings showed that mDFPS among women living in the urban area varied substantially by country, with West and Central Africa countries presenting the lowest mean coverage across the regions. Large within-country wealth inequalities in mDFPS were found for most countries included in our analyses, with a pattern of lower mDFPS coverage among the poorest women compared to the wealthiest. Differences in the type of contraceptive method used were also observed with an overall pattern of lower use of long-acting and permanent methods among the urban poor, but a few countries showed the opposite pattern. Our ecological analyses indicated that a higher proportion of informal settlements tend to be related to lower mDFPS coverage and higher relative wealth-related inequalities.

African countries are experiencing a rapid urban expansion largely due to natural population growth (4, 28). The rapid urbanization in African settings, concomitant with poor governance and slow economic growth, has led to informal settlements that are overcrowded and with poor infrastructure (10). There is evidence that several health conditions in these settings are better than among poor-rural women, however, it is frequently much worse than among urban non-informal settlements (29, 30).

In agreement with our findings, other studies have also found lower coverage of family planning services among informal settlements residents than urban non-informal settlements residents, where common barriers to modern contraceptive use are cost of method and lack of knowledge and access to modern contraceptives (30–33). There is also evidence of short birth intervals and higher prevalence of adolescent motherhood among informal settlement dwellers than among the non-informal settlements (30). In addition to financial barriers, adolescents tend to deal with additional barriers to access contraception, such as community disapproval of sexual activity during adolescence and social norms pressuring them to prove their fertility soon as they get married (34). Living in informal settlements have been contextualized as a mediator in the relationship between socioeconomic factors (such as wealth and education) and sexual and reproductive health outcomes (31). Previous studies also indicate a higher proportion of risky sexual behaviors in adolescents who grow up in informal settlements, such as multiple sexual partners, first sexual intercourse in early age, and unprotected sex.

Urban informal settlements are often neglected settings, with important limitations in relation to health services provision (33). Our findings support this evidence, since the higher the proportion of informal settlements, the lower the mDFPS coverage. Supply-side interventions may be extremely relevant in increasing family planning coverage in urban African settings, especially among non-users, when based on a rights-based approach and when considering the contexts where these women are located and their particular needs (13).

Our findings revealed important inequalities in mDFPS between and within the countries studied. Sudan, Angola, Central African Republic, the Democratic Republic of the Congo, and Mauritania, for instance, presented the lowest national mDFPS coverage among urban women and presented large wealth inequalities in mDFPS with the urban poor faring worst. In addition to the out-of-pocket payment that may be required to get contraceptives (35), there is evidence of a lower approval of family planning by the husband among poorer couples (36). Both factors can be contributing reasons for lower mDFPS coverage among the poorest. Prior studies also documented significant gaps in mDFPS coverage in these countries according to area of residence, level of education, religion, and age (18, 35). Besides the current unfavorable scenario in terms of inequalities, slow improvements in national family planning coverage have also been reported for these countries in the last years (14, 37).

Chad and Central African Republic had the lowest mDFPS coverage. Both are among the poorest countries in the world and have large gender inequalities, leading the rank of child marriage rates and with high levels of illiterate girls (38). In addition to their cultural norms, the high poverty rates in these countries push girls to early marriage to reduce family expenses and increase their assets, through the dowry (38). Besides that, the economy in these countries is highly reliant on subsistence farming, which favors bigger families and may partially explains the persistent lower demand for family planning in comparison to other African countries (39, 40). Even with the lower demand for family planning, both countries still have a high proportion of demand for family planning unsatisfied. Chad presented the smallest mDFPS coverage among all the countries included in our analyses as well as enormous wealth inequalities, leaving behind the poorest urban women. Central African Republic similarly presented a small national coverage of mDFPS, with huge gaps in mDFPS between the poorest and wealthiest women in urban areas. Our findings support projections indicating that universal coverage of family planning services would only be reached in 2076 in Central African Republic, and in 2100 in Chad (41).

Sustainable mDFPS coverage depends on the availability of a range of contraceptive methods, guaranteeing different options depending on women's needs and wants (42). Long-acting reversible methods have lower failure rates. They are cost-effective and do not require frequent attention by their users (43). However, long-acting reversible methods require special conditions such as a qualified health worker, appropriate place to be inserted, and medical follow-up after procedure due to risks of uterine perforation, infection, or expulsion. The additional requirements for safe provision of long-acting reversible methods may explain the more frequent use of short-acting reversible methods in many African countries (28, 44). Not surprisingly, the share of short-acting reversible methods was higher among the poorest urban women in several countries. The unavailability of long-acting reversible methods in informal settings can result in limited choice and contribute to lower mDFPS among the urban poor (32).

Our study is not free of limitations. The analyses about informal settlements are based on a common household-centered definition of informal settlement, where most of the classification elements are basic household living conditions instead of characteristics of a neighborhood or a larger area. Therefore, it represents an additional metric of wealth inequalities, but it lacks the important aspect of the environment. A more detailed definition of informal settlements, including the neighborhood in which the household is located, should be adopted to take into consideration the influence of contextual factors on sexual and reproductive health indicators. The analyses presented here were limited to wealth-related inequalities; however, other socioeconomic factors such as education and age could also present large mDFPS coverage gaps. Inequalities regarding these factors should be analyzed individually and concurrently with other factors using double stratification analyses. Despite of these limitations, our findings shed new light on the need to consider the intersectionality of the several axes of inequality and on the presented intra-urban wealth inequalities in mDFPS.

## Conclusion

We showed that mDFPS coverage among urban populations is highly variable across Africa and most countries presented significant wealth inequalities with the lowest coverage among poorer urban dwellers compared to their wealthier counterparts. Our findings highlight the need for greater investments to improve access to and use of family planning services in urban poor contexts. Our findings also underscore the value of future research using individual data on countries where inequalities were found.

The interconnection between rapid urbanization, poverty, and sexual and reproductive health is often ignored in the global development agenda as living in urban areas is often assumed to mean better access to health services and better health outcomes. However, this view fails to consider the significant vulnerability of the poorest urban women, who live in precarious conditions. The rapid urbanization occurring in many African countries with weak economies has led to the precipitous growth of informal settlements. In these urban poor contexts, it is important to consider programs such as asset building and economic empowerment strategies that also address the socio-economic drivers of poor sexual and reproductive health outcomes (45).

## Data Availability Statement

The original contributions presented in the study are included in the article/Supplementary Materials, further inquiries can be directed to the corresponding author/s.

## Author Contributions

FH, CC, and CB proposed the idea, outlined the methods to be used in the study, and wrote the initial draft of the manuscript. FH and CB analyzed the data. AB supervised all statistical analysis. All authors contributed to the article, revised the final version and approved the submitted version.

## Conflict of Interest

The authors declare that the research was conducted in the absence of any commercial or financial relationships that could be construed as a potential conflict of interest.

## Publisher's Note

All claims expressed in this article are solely those of the authors and do not necessarily represent those of their affiliated organizations, or those of the publisher, the editors and the reviewers. Any product that may be evaluated in this article, or claim that may be made by its manufacturer, is not guaranteed or endorsed by the publisher.

## References

[1] Jatana N Currie A. Hitting the Targets: The Case for Ethical and Empowering Population Policies to Accelerate Programs Towards the Sustainable Development Goals. (2020). Available online at: https://populationmatters.org/sites/default/files/Hitting%20the%20Targets%20-%20Population%20and%20the%20SDGs.pdf.

[2] OECD/SWAC. Africa's Urbanisation Dynamics 2020. OECD. West African Studies (2020). p. 145.

[3] Nation U. World urbanization prospects: an alternative to the UN model of projection compatible with the mobility transition theory. Demogr Res. (2018). 12:197–236. 10.4054/DemRes.2005.12.9

[4] Fotso JC Speizer IS Mukiira C Kizito P Lumumba V. Closing the poor-rich gap in contraceptive use in urban Kenya: are family planning programs increasingly reaching the urban poor? Int J Equity Health. (2013) 12:1–10. 10.1186/1475-9276-12-71 23978064 PMC3847584

[5] Yadav K Agarwal M Shukla M Singh JV Singh VK. Unmet need for family planning services among young married women (15–24 years) living in urban slums of India. BMC Womens Health. (2020) 20:187. 10.1186/s12905-020-01010-9 32883262 PMC7469334

[6] Feinberg R Fay M. the urban poor in latin America. Foreign Aff. (2005) 84:148. 10.2307/20031811

[7] Magadi M. Maternal and child health among the urban poor in Nairobi, Kenya. Etud Popul Afric. (2004). 19:171–90. Available online at: http://www.bioline.org.br/pdf?ep04041

[8] Ziraba AK Madise N Mills S Kyobutungi C Ezeh A. Maternal mortality in the informal settlements of Nairobi city: what do we know? Reprod Health. (2009) 6:6. 10.1186/1742-4755-6-6 19386134 PMC2675520

[9] Fotso JC. Urban-rural differentials in child malnutrition: trends and socioeconomic correlates in sub-Saharan Africa. Heal Place. (2007) 13:205–23. 10.1016/j.healthplace.2006.01.004 16563851

[10] Mberu B Mumah J Kabiru C Brinton J. Bringing sexual and reproductive health in the urban contexts to the forefront of the development agenda: the case for prioritizing the urban poor. Matern Child Health J. (2014) 18:1572–7. 10.1007/s10995-013-1414-7 24352624 PMC4152622

[11] Tsui AO McDonald-Mosley R Burke AE. Family planning and the burden of unintended pregnancies. Epidemiol Rev. (2010) 32:152–74. 10.1093/epirev/mxq012 20570955 PMC3115338

[12] Beguy D Mumah J Gottschalk L. Unintended pregnancies among young women living in urban slums: evidence from a prospective study in Nairobi City, Kenya. PLoS ONE. (2014) 9:e101034. 10.1371/journal.pone.0101034 25080352 PMC4117474

[13] Winston J Calhoun LM Corroon M Guilkey D Speizer I. Impact of the urban reproductive health initiative on family planning uptake at facilities in Kenya, Nigeria, and Senegal. BMC Womens Health. (2018) 18:9. 10.1186/s12905-017-0504-x 29304793 PMC5756340

[14] Hellwig F Coll CVN Ewerling F Barros AJ. Time trends in demand for family planning satisfied : analysis of 73 countries using national health surveys over a 24-year period. J Glob Health. (2019). 9:020423. 10.7189/jogh.09.020423 31673339 PMC6820067

[15] Countdown to 2030: tracking progress towards universal coverage for reproductive maternal newborn and child health. Lancet. (2018). 391:P1538–48. 10.1016/S0140-6736(18)30104-1 29395268

[16] Countdown to 2030. Tracking Progress Towards Universal Coverage for Women's, Children's and Adolescents' Health: The 2017 Report. (2017). p. 260. Available online at: http://countdown2030.org/pdf/Countdown-2030-complete-with-profiles.pdf (accessed May 18, 2021).

[17] Benson A Calhoun L Corroon M Gueye A Guilkey D Kebede E. The Senegal urban reproductive health initiative: a longitudinal program impact evaluation. Contraception. (2018) 97:439–44. 10.1016/j.contraception.2018.01.003 29352973 PMC5948164

[18] Ewerling F Victora CG Raj A Coll CVN Hellwig F Barros AJD. Demand for family planning satisfied with modern methods among sexually active women in low- and middle-income countries: who is lagging behind? Reprod Health. (2018). 15:42. 10.1186/s12978-018-0483-x 29510682 PMC5840731

[19] Hubacher D Trussell J. A definition of modern contraceptive methods. Contraception. (2015) 92:420–1. 10.1016/j.contraception.2015.08.008 26276245

[20] Rutstein SO. The DHS Wealth Index: Approaches for Rural and Urban Areas. DHS Working Papers No 60. Macro International Inc., Calverton, MD (2008).

[21] Rutstein SO. The DHS Wealth Index: Approaches for Rural and Urban Areas. DHS Working Papers No 60. Macro International Inc., Calverton, MD. (2008).

[22] UNICEF. UNICEF Regional Classifications (2017). Available online at: https://data.unicef.org/regionalclassifications/ (assessed May 22, 2021).

[23] Donnell OO Wagstaff A Lindelow M. Analyzing Health Equity Using Household Survey Data: A Guide to Techniques and Their Implementation. Washington, DC: World Bank.

[24] Barros AJD Victora CG. Measuring Coverage in MNCH: determining and interpreting inequalities in coverage of maternal, newborn, and child health interventions. PLoS Med. (2013) 10:e1001390. 10.1371/journal.pmed.1001390 23667332 PMC3646214

[25] UN-Habitat. Training Module: Adequate Housing and Slum Upgrading. Nairobi: (2018).

[26] Bank W. World Bank Open Data: Free and Open Access to Global Development Data. Available online at: https://data.worldbank.org/ (assessed February 10, 2021).

[27] Harrell FE. Regression Modeling Strategies: With Application to Linear Models, Logistic Regression, and Survival Analysis. New York: Springer Science and Business Media. (2001). 10.1007/978-1-4757-3462-1

[28] Ouedraogo L Habonimana D Nkurunziza T Chilanga A Hayfa E. Towards achieving the family planning targets in the African region : a rapid review of task sharing policies. Reprod Health. (2021). 18:22. 10.1186/s12978-020-01038-y 33485339 PMC7825212

[29] Fink G Günther I Hill K. Slum residence and child health in developing countries. Demography. (2014) 51:1175–97. 10.1007/s13524-014-0302-0 24895049

[30] Mberu BU Haregu TN Kyobutungi C Ezeh AC. Health and health-related indicators in slum, rural, and urban communities : a comparative analysis. Glob Health Action. (2016) 9:10.3402/gha.v9.33163. 10.3402/gha.v9.33163 27924741 PMC5141369

[31] Wado YD Bangha M Kabiru CW Feyissa GT. Nature of, and responses to key sexual and reproductive health challenges for adolescents in urban slums in sub-Saharan Africa : a scoping review. Reprod Heal. (2020). 17:149. 10.1186/s12978-020-00998-5 32998741 PMC7526107

[32] Ochako R Izugbara C Okal J Askew I Temmerman M. Contraceptive method choice among women in slum and non-slum communities in Nairobi, Kenya. BMC Womens Health. (2016) 16:35. 10.1186/s12905-016-0314-6 27405374 PMC4941019

[33] Hazarika I. Women' s reproductive health in slum populations in India : evidence from NFHS-3. J Urban Heal. (2009) 87:264–77. 10.1007/s11524-009-9421-0 PMC2845837 20148311

[34] De Vargas Nunes Coll C Ewerling F Hellwig F De Barros AJD. Contraception in adolescence: the influence of parity and marital status on contraceptive use in 73 low-and middle-income countries. Reprod Health. (2019). 16:21. 10.1186/s12978-019-0686-9 30791914 PMC6383262

[35] Kandala N-B Lukumu FK Mantempa JN Kandala JD Chirwa T. Disparities in modern contraception use among women in the Democratic Republic of Congo: a cross-sectional spatial analysis of provincial variations based on household survey data. J Biosoc Sci. (2015) 47:345–62. 10.1017/S0021932014000212 24911333

[36] Prata N Bell S Fraser A Carvalho A Neves I. Partner support for family planning and modern contraceptive use in Luanda, Angola. Afr J Reprod Health. (2017). 21:35–48. 10.29063/ajrh2017/v21i2.5 29624938

[37] Alkema L Kantorova V Menozzi C Biddlecom A. National, regional, and global rates and trends in contraceptive prevalence and unmet need for family planning between 1990 and 2015: a systematic and comprehensive analysis. Lancet. (2013). 381:1642–52. 10.1016/S0140-6736(12)62204-1 23489750

[38] Girls Not Brides. Child Marriage. Available online at: https://www.girlsnotbrides.org/ (accessed February 19, 2021).

[39] Cahill N Sonneveldt E Stover J Weinberger M Williamson J Wei C. Modern contraceptive use, unmet need, and demand satisfied among women of reproductive age who are married or in a union in the focus countries of the Family Planning 2020 initiative : a systematic analysis using the Family Planning Estimation Tool. Lancet. (2020) 391:870–82. 10.1016/S0140-6736(17)33104-5 29217374 PMC5854461

[40] Götmark F. Human fertility in relation to education, economy, religion, contraception, and family planning programs. BMC Public Health. (2020) 7:1–17. 10.1186/s12889-020-8331-7 32087705 PMC7036237

[41] Cahill N Alkema L. What increase in modern contraceptive use is needed in FP2020 countries to reach 75 % demand satisfied by 2030? An assessment using the Accelerated Transition Method and Family Planning Estimation Model. Gates Open Res. (2020). 4:113. 10.12688/gatesopenres.13125.1 33117964 PMC7578409

[42] Cleland J Bernstein S Ezeh A Faundes A Glasier A Innis J. Family planning: the unfinished agenda. Lancet. (2006). 368:1810–27. 10.1016/S0140-6736(06)69480-4 17113431

[43] Gomez R De Leon P Ewerling F Serruya SJ Silveira MF Sanhueza A. Contraceptive use in Latin America and the Caribbean with a focus on long-acting reversible contraceptives : prevalence and inequalities in 23 countries. Lancet Glob Heal. (2019) 7:e227–35. 10.1016/S2214-109X(18)30481-9 30683240 PMC6367565

[44] Lince-deroche N Hendrickson C Moolla A Kgowedi S Mulongo M. Provider perspectives on contraceptive service delivery : findings from a qualitative study in Johannesburg, South Africa. BMC Health Serv Res. (2020). 9:128. 10.1186/s12913-020-4900-9 32085756 PMC7035764

[45] Tsui AO Brown W Li Q. Contraceptive practive in Sub-Saharan Africa. Popul Dev Rev. (2017) 43:166–91. 10.1111/padr.12051 29081552 PMC5658050

