# Supplementary material for: Assessing Wealth-Related Inequalities in Demand for Family Planning Satisfied in 43 African Countries
Source: Front Glob Womens Health. 2021 Jul 26;2:674227. doi: 10.3389/fgwh.2021.674227 (PMC8594043; doi:10.3389/fgwh.2021.674227)
Supplement: Supplementary file 1 [file Table_1.docx]

## Supplementary material

Supplementary Table 1 - Model quality adjustment criteria for urban slum.

| **Urbanization variable** | **mDFPS** | | | **CIX** | | |
| --- | --- | --- | --- | --- | --- | --- |
|  | **Number of knots** | **AIC** | **BIC** | **Number of knots** | **AIC** | **BIC** |
| **Population living in slums** | 3 | 361.17 | 366.39 | 3 | 276.81 | 282.02 |
|  | 4 | 361.36 | 368.31 | 4 | 275.59 | 282.54 |
|  | 5 | 363.31 | 372.00 | 5 | 276.80 | 285.49 |
|  | 6 | 365.23 | 375.66 | 6 | 279.13 | 289.55 |
|  | 7 | 367.16 | 379.32 | 7 | 280.18 | 292.34 |
| AIC: Akaike's Information Criterion; BIC: Bayesian Information Criterion; CIX: concentration index; mDFPS: demand for family planning satisfied with modern methods | | | | | | |
